# Transcutaneous assessment of glomerular filtration rate in unanesthetized rats using a small animal imager: impact on arterial pressure, heart rate, and activity

**DOI:** 10.14814/phy2.12723

**Published:** 2016-10-10

**Authors:** Lucinda M. Hilliard, Kate M. Denton

**Affiliations:** ^1^ Cardiovascular ProgramMonash Biomedicine Discovery Institute and Departments of PhysiologyMonash UniversityMelbourneVictoria3800Australia

**Keywords:** Arterial pressure, FITC‐sinistrin clearance, glomerular filtration rate, kidney function, rat

## Abstract

Transcutaneous assessment of fluorescein isothiocyanate (FITC)‐sinistrin clearance using a small animal imager has recently been validated as an accurate method for the measurement of glomerular filtration rate (GFR) in freely moving rats. This technique involves a brief, light period of anesthesia during which the imager is adhered to the rat and FITC‐sinistrin is administered. The rat is then moved to an experimental chamber where it is housed unrestrained for a 2‐h data collection period. This study assessed the impact of the experimental protocol on mean arterial pressure (MAP), heart rate, and locomotor activity in adult Sprague Dawley rats using radiotelemetry given that anesthesia and stress are known to affect arterial pressure and kidney function. These data were compared with time‐equivalent measurements made in the same rats at rest. MAP was low following anesthesia, but increased within 15 min and remained stable thereafter. Heart rate was not affected by the GFR protocol. Locomotor activity increased following anesthesia before decreasing to relatively low levels during the final 75‐min, the approximate period from which GFR is calculated. Moreover, MAP, heart rate, and locomotor activity during the final 75‐min of the data collection period were not different to that observed during an equivalent time period at baseline. Taken together, our findings suggest that this recently developed minimally invasive procedure for the measurement of GFR in unanesthetized rats does not negatively impact arterial pressure, heart rate, or locomotor activity. Thus, it is likely to be a valuable implement for acquiring serial measurements of GFR in unanesthetized rats.

## Introduction

Investigations into renal function in health and disease in small laboratory animals, including rats, contribute significantly to our understanding of renal physiology and pathophysiology in humans. Infusion‐based methods for assessment of plasma or urine clearance of exogenous renal markers, especially inulin, have long been recognized as the gold standard for accurate measurement of glomerular filtration rate (GFR) in the laboratory rat (Beierwaltes et al. 2013). However, such methods are invasive. They are often performed under anesthesia or in conscious rats several hours after surgical implantation of catheters. Moreover, they commonly require surgical preparation and repeated blood and/or urine sampling, and can induce stress and hypovolemia; all factors that can affect arterial pressure and kidney function and thus impede accurate determination of GFR (Walker et al. 1983; Burchardi and Kaczmarczyk 1994; Fusellier et al. 2007; Schock‐Kusch et al. 2009). Furthermore, such techniques limit or prevent the ability of investigators to perform repeated measurements in the same animals over time. Notably, various approaches for transcutaneous measurement of GFR based on the elimination kinetics of fluorescent exogenous markers have been developed in recent years (Dorshow et al. 1998; Rabito et al. 2005; Schock‐Kusch et al. 2009; Wang et al. 2010). However, while these methods negate the requirement for blood and urine collection and facilitate the real‐time determination of GFR, invasive surgery is required and data were collected from rats during anesthesia.

In light of these shortcomings, a transcutaneous approach for measurement of GFR in unanesthetized, freely moving rats was developed and validated in recent years using fluorescein isothiocyanate (FITC)‐sinistrin, a fluorescent‐labeled exogenous marker of GFR (Schock‐Kusch et al. 2011). This technique involves measurement of the elimination kinetics of FITC‐sinistrin following its injection into the peripheral circulation. This is achieved using a small animal imager (noninvasive clearance [NIC]‐Kidney device; Mannheim Pharma & Diagnostics GmbH, Mannheim, Germany), programmed to detect fluorescence intensity, which is adhered to a depilated region on the back of the rat (Schock‐Kusch et al. 2011). Given the minimally invasive nature of this technique, serial measurements of GFR can be taken in the same rat within a short time frame to assess time‐ and treatment‐dependent changes in kidney function (Cowley et al. 2013; Evans et al. 2015).

The procedure for the application of the NIC‐Kidney device in rats involves a brief, light gas anesthesia period during which a small patch of skin is depilated and the miniaturized device is attached. FITC‐sinistrin is then administered, commonly via the tail vein, while the rat is still under anesthesia or via a previously catheterized external jugular or femoral vein once the rat is awake (Schock‐Kusch et al. 2011; Cowley et al. 2013; Evans et al. 2015; Peters et al. 2015). The rat is subsequently housed unrestrained in an experimental chamber for ~2 h. Finally, the acquired data were used to generate an elimination kinetics curve of FITC‐sinistrin. The half‐life of FITC‐sinistrin can be used as a valid measure of kidney function. Alternatively, a pre‐established empirically derived conversion factor has been validated for the calculation of GFR from the FITC‐sinistrin half‐life in adult rats (Schock‐Kusch et al. 2009).

Although light anesthesia is administered to rats during the preparatory period, the half‐life of FITC‐sinistrin is calculated from the single exponential excretion phase of the elimination kinetics curve which, in adult rats, usually begins ~45 min after the administration of FITC‐sinistrin (Schock‐Kusch et al. 2009) and subsequent removal of anesthesia. Furthermore, the FITC‐sinistrin clearance data were acquired from unrestrained, freely moving rats, thereby minimizing the likely impact of stress on measurement of GFR. Therefore, to investigate these factors in further detail, we assessed the impact of the preparation protocol on arterial pressure, heart rate, and locomotor activity using radiotelemetry. Furthermore, we monitored these variables across the duration of the GFR study and compared this to equivalent time periods on the day prior during which the rats were resting in their home cage.

## Methods

### Animals

Male Sprague Dawley rats (*n* = 8) were obtained from Monash Animal Research Platform (Monash University, Melbourne, Victoria, Australia). Rats were housed individually under standard laboratory conditions (12‐h light/dark cycle at a temperature of 21°C) and were fed a sodium‐controlled diet (0.25% sodium chloride; Specialty Feeds, Glen Forrest, Western Australia, Australia) and water ad libitum. Experiments were approved by the Monash University, School of Biomedical Sciences Animal Ethics Committee and were performed in accordance with the Australian Code of Practice for the Care and Use of Animals for Scientific Purposes. Rats were acclimatized to these conditions prior to the commencement of the study protocol.

### Mean arterial pressure and GFR

At 22 weeks of age, rats were anesthetized (isoflurane; 2–5% O_2_) and a radiotelemetry probe (PA‐C40; Data Sciences International, St. Paul, MN) was implanted into the abdominal aorta for the continuous measurement of arterial pressure (systolic, diastolic, and mean), and heart rate, as described previously (Sampson et al. 2008).

**Figure 1:**
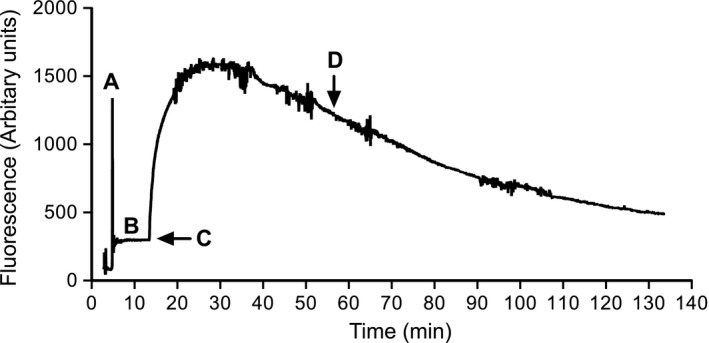
Representative transcutaneous disappearance curve of FITC‐sinistrin. The time course of the GFR protocol is shown. (A) The signal detected prior to attachment of the device to the rat, (B) the baseline measurement period (background fluorescence of the skin), (C) the time of the intravenous FITC‐sinistrin injection, and (D) the approximate start of exponential excretion of FITC‐sinistrin (~45 min postinjection of FITC‐sinistrin). FITC, fluorescein isothiocyanate; GFR, glomerular filtration rate.

After a 10‐day recovery period, basal arterial pressure, heart rate, and locomotor activity was recorded. These data were scheduled to be recorded as 10 sec averages every 10 min. The following day, GFR was measured in rats via the transcutaneous clearance of FITC‐sinistrin using a NIC‐Kidney device (with internal memory; Mannheim Pharma & Diagnostics GmbH, Mannheim, Germany). This was performed around the same time of day in all rats (between 9 am and 12 pm; near the beginning of the light cycle) in order to minimize the impact of circadian variation in GFR. Rats were lightly anesthetized for ~10 min or less (2–2.5% v/v isoflurane). During this time, the device was turned on via connection to a lithium polymer rechargeable battery, and then attached to a depilated region on the back of the rat using a double‐sided adhesive patch and adhesive tape. Then, following a ~3‐ to 5‐min baseline period, a bolus of FITC‐sinistrin (3 mg/100 g made up in 0.9% sodium chloride solution; Fresenius Kabi Austria GmbH, Linz, Austria) was administered via the tail vein. The rat was then moved to an experimental chamber (containing bedding chips from the rat's home cage), which was then placed on a telemetry receiver for 2 h, and the rat quickly awakened from the anesthesia. These chambers were used in place of the rats’ home cages since they were taller in size and therefore minimized the risk of dislodgment of the device from the back of the rat, particularly when the animal was awakening from anesthesia and moving around the cage. Thereafter, arterial pressure, heart rate, and locomotor activity recordings were scheduled to be continuously recorded. At the end of the 2‐h recording period, rats were lightly anesthetized and the device was removed. The acquired data were subsequently analyzed using NIC‐Kidney device partner software (MPDlab v1.0, Mannheim Pharma & Diagnostics GmbH). The software generates the elimination kinetics curve of FITC‐sinistrin from which elimination half‐life determinations were calculated from the single exponential excretion phase of the curve (approximate last 75 min of the 2‐h recording period postinjection of FITC‐sinistrin; as shown in Fig. 1) using a one‐compartment model. Moreover, the half‐life was then used to calculate GFR using a empirically derived conversion factor for rats using the following formula: GFR (mL/min per 100 g BW) = 31.26 mL/100 g BW ÷ half‐life of FITC‐sinistrin (min) (Schock‐Kusch et al. 2009). All rats had access to food and water except during the 2‐h GFR measurement period.

To determine whether the GFR experimental protocol led to a significant change in arterial pressure, heart rate, or locomotor activity, these variables were monitored continuously across the 2‐h data collection period, which included the arousal of rats from light anesthesia. Furthermore, the average measurements of arterial pressure, heart rate, and locomotor activity during the 75‐min period from which the elimination half‐life of FITC‐sinistrin was calculated were compared to the average of these variables at baseline during the equivalent 75‐min window (9.45 am to 11.00 am) when the animals were in their home cage on the day prior. In addition, the 10‐min interval recordings of these variables on the day prior to the GFR measurement between 9.00 am and 11.00 am were compared to the equivalent 10‐min interval recordings made during the 2‐h GFR experimental protocol.

### Statistical analyses

Data are presented as mean ± standard error of the mean (SEM). Arterial pressure, heart rate, and locomotor activity data during the 75‐min GFR measurement period and during an equivalent 75‐min period at rest were analyzed using Student's paired *t* tests. Two‐tailed *P* values ≤0.05 were considered statistically significant.

## Results

A representative transcutaneous disappearance curve of FITC‐sinistrin, which identifies the time course of our GFR protocol is provided in Figure 1. Half‐life of sinistrin and GFR averaged 35.9 ± 1.2 min and 0.87 ± 0.03 mL/min per 100 g BW, respectively.

**Figure 2:**
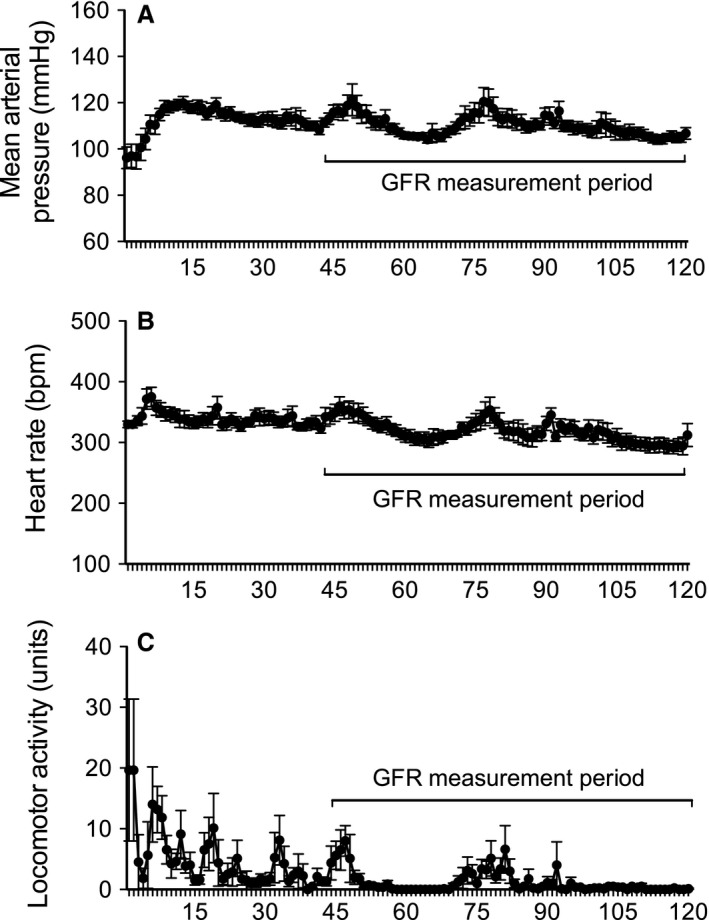
Continuous recordings of (A) mean arterial pressure, (B) heart rate, and (C) locomotor activity over the 2‐h GFR measurement period. The 75‐min window from which the half‐life of FITC‐sinistrin was derived is highlighted. All data are presented as mean ± SEM. *n* = 8. GFR, glomerular filtration rate; FITC, fluorescein isothiocyanate.

**Figure 3:**
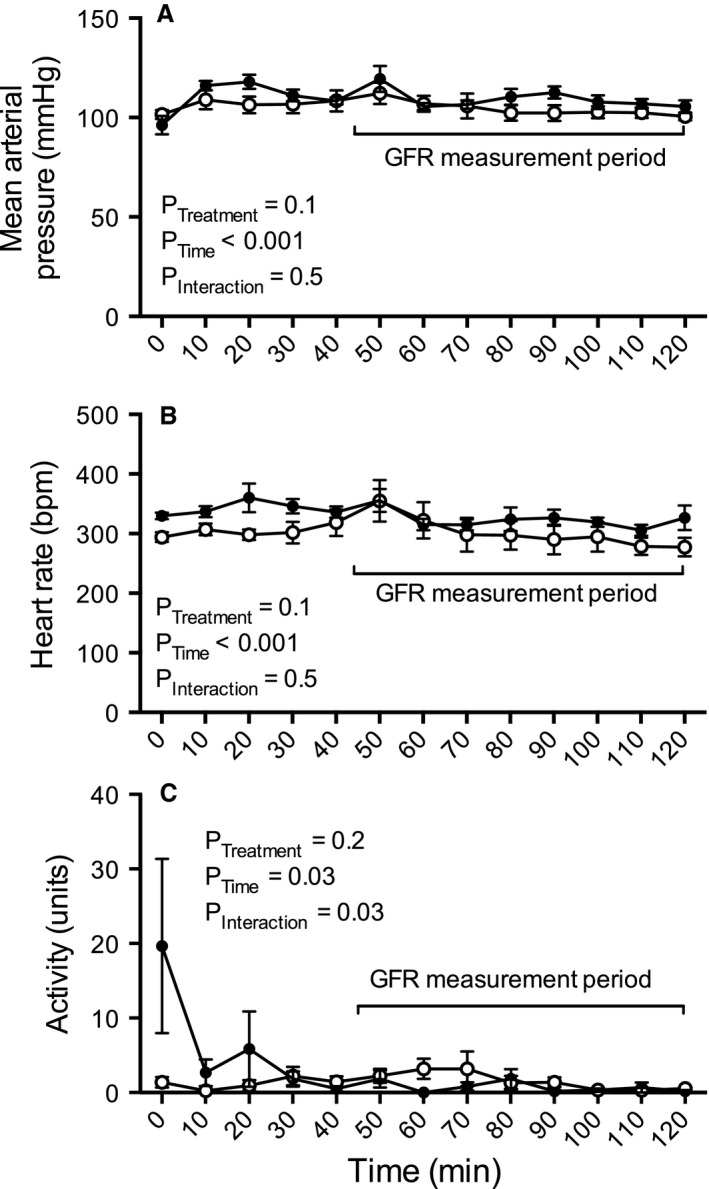
Ten‐minute interval measurements of (A) mean arterial pressure, (B) heart rate, and (C) locomotor activity over the 2‐h GFR measurement period (closed circles) as compared to the equivalent 2‐h period during the final day of the baseline measurement period (denoted as baseline; open circles). The 75‐min window from which the half‐life of FITC‐sinistrin was derived is highlighted. All data are presented as mean ± SEM. *n* = 6. GFR, glomerular filtration rate; FITC, fluorescein isothiocyanate.

**Figure 4:**
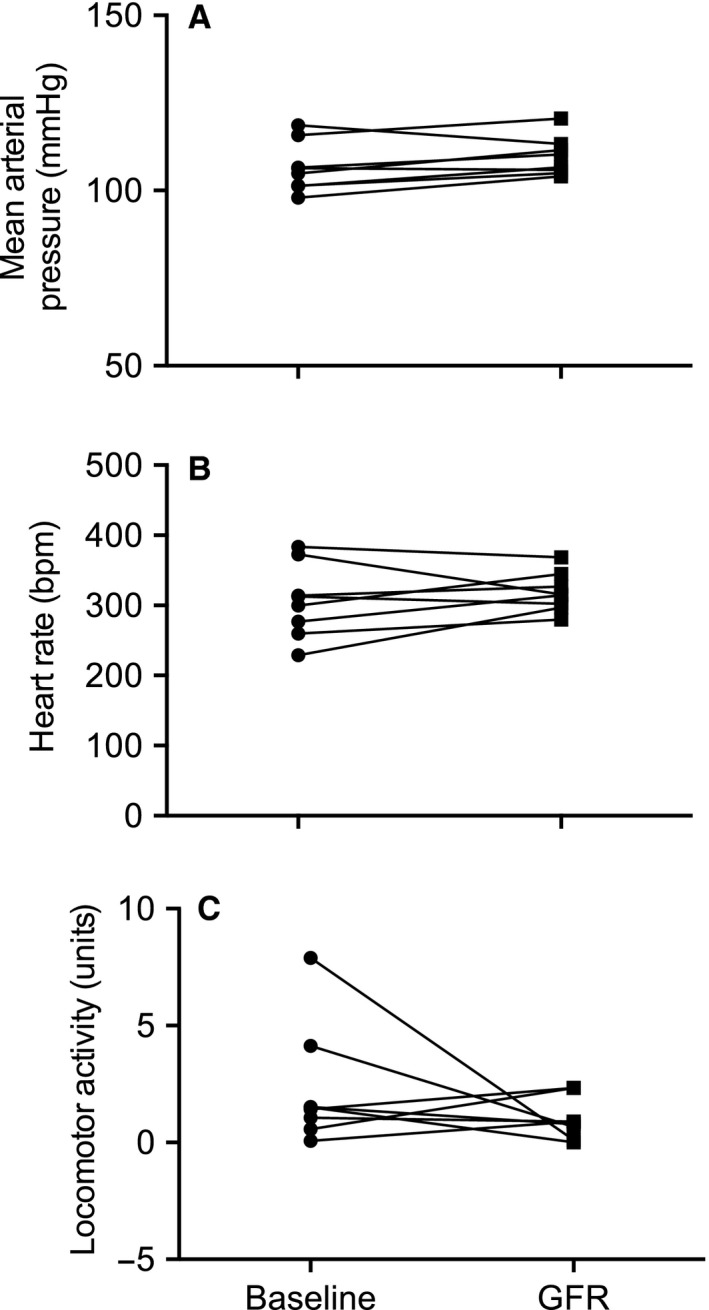
(A) Average mean arterial pressure, (B) heart rate, and (C) locomotor activity during 75‐min measurement of glomerular filtration rate (GFR) (denoted as GFR) as compared to equivalent 75‐min period during the final day of the baseline measurement period (denoted as baseline). All data are presented as mean ± SEM. *n* = 8.

Following recovery from the brief, light isoflurane anesthesia in preparation for the transcutaneous measurement of FITC‐sinistrin clearance, mean arterial pressure (MAP) was low, but quickly increased plateauing within 15 min (Fig. 2A). Thereafter, MAP remained relatively constant across the remainder of the 2‐h recording period. Moreover, there was no difference in MAP during the 75‐min period from which GFR is calculated as compared to during an equivalent 75‐min window of the baseline measurement period (Figs. 3A, 4A). Comparable observations were documented for both systolic and diastolic pressure (data not shown). On average MAP was 3.0 ± 1.4 mmHg greater during the GFR measurement period as compared to during baseline.

There was no significant impact of the GFR protocol on heart rate (Fig. 2A). Heart rate remained relatively constant across the 2‐h recording period. Furthermore, during the 75‐min GFR measurement period heart rate was similar to that observed during the equivalent time period on the day prior (Figs. 3B, 4B). On average, heart rate was 13 ± 14 bpm higher during the GFR measurement period as compared to during baseline.

Locomotor activity was also not significantly affected by the GFR protocol. Following light anesthesia prior to measurement of GFR, locomotor activity increased before decreasing to relatively low levels within 45 min (Fig. 2C). Moreover, there was no difference in locomotor activity during the 75‐min GFR measurement period as compared to during an equivalent 75‐min window of the baseline measurement period (Figs. 3C, 4C). On average locomotor activity was 3.9 ± 1.3 units lower during the GFR measurement period as compared to during baseline.

## Discussion

The novel finding of the current study was that the described procedure for the measurement of GFR in unanesthetized, freely moving rats using a NIC‐Kidney device has a negligible impact on arterial pressure, heart rate, or locomotor activity during the period from which GFR is determined. All three variables quickly stabilized following the preparatory phase of the experimental protocol during which rats were lightly anesthetized. Moreover, arterial pressure, heart rate, and locomotor activity remained steady thereafter, analogous to that observed in the same rats on the day prior when undisturbed in their home cage. Our findings therefore provide further justification for the application of this technique as a valuable tool for studies in which repeated measurements of GFR in unanesthetized rats are required.

In proof‐of‐principle experiments, Schock‐Kusch and colleagues previously compared simultaneous measurements of transcutaneous (using a NIC‐Kidney device) and plasma elimination kinetics of FITC‐sinistrin in freely moving rats with normal and reduced kidney function (Schock‐Kusch et al. 2011). Importantly, their results showed highly analogous elimination half‐lives and GFR measurements across all rat models used, thus providing critical validations for the application of this technique for repeated, reliable measurements of GFR in rats. Moreover, in other studies Cowley and colleagues demonstrated the ability of this technique to provide reproducible measurements of GFR in rats across sequential days in two separate studies (Cowley et al. 2013; Evans et al. 2015). This included a time–control cohort of rats in which GFR was measured seven times over a ~21‐day period and consistent GFR values were obtained (Cowley et al. 2013).

Importantly, our findings extend these observations by demonstrating a negligible effect of the experimental protocol on arterial pressure, heart rate, and locomotor activity. Rats recovered quickly from the preparatory period for the application of the NIC‐Kidney device and injection of FITC‐sinistrin, during which light gas anesthesia was administered. Arterial pressure, heart rate, and locomotor activity remained similar to baseline levels thereafter for the remainder of the 2‐h data collection period. Furthermore, GFR measurements were acquired which are comparable with our previous observations in age‐matched rats as determined via assessment of clearance of inulin, the gold standard of GFR measurement (Zimanyi et al. 2006).

Consistent with our own observations, Cowley et al. reported arterial pressure measurements similar to basal levels during transcutaneous measurement of FITC‐sinistrin using a NIC‐Kidney device in studies performed in Dahl salt‐sensitive hypertensive rats (Cowley et al. 2013). However, there were several key differences in study design and the time course of the GFR preparation protocol adopted by Cowley and colleagues as compared to our own. First, arterial pressure measurements taken from rats undergoing the transcutaneous GFR protocol were compared to measurements obtained from a separate cohort of rats. Second, the anesthesia period in the study by Cowley et al. was less than 5 min since it was only used for depilation of the skin and subsequent attachment of the NIC‐Kidney device. FITC‐sinistrin was administered to rats at least 40–50 min following the anesthesia period via an existing femoral artery catheter thereby presumably allowing >85 min for rats to recover from the light anesthesia. In comparison, a technical advantage of our study design given the nature of our aims was that our rats were instrumented for simultaneous measurement of arterial pressure and GFR. This consequently allowed us to perform within‐animal comparisons of arterial pressure across time. Furthermore, we administered FITC‐sinistrin during the anesthesia period. Thus, we were able to ascertain the impact of the GFR protocol on MAP, heart rate, and locomotor activity with a shorter recovery time (~45 min). Of course, it cannot be ruled out that compensatory mechanisms are activated during the brief period of light isoflurane anesthesia, such as the renin–angiotensin system, that subsequently contribute to the normalization of arterial pressure during the GFR measurement period, which could also affect GFR. It would therefore be of interest in future studies to compare plasma renin activity at baseline and at the commencement of the GFR measurement period. If differences in plasma renin activity were observed it would be important to allow more time for recovery from anesthesia before measurements of FITC‐sinistrin elimination half‐life are made.

Furthermore, it should be recognized that we performed measurements of FITC‐sinistrin clearance during the resting period (light cycle; between 9 am and 12 pm) in order to minimize the impact of circadian variation in GFR. It is likely that this time choice contributed to the lack of effect of the 2‐h experimental protocol on arterial pressure. In this context, rats were commonly observed to rest or sleep during the time period from which the half‐life of FITC‐sinistrin was determined, as also demonstrated by our locomotor activity data. This is consistent with their behavior during the same time period when left undisturbed in their home cage. We therefore suggest that measurement of FITC‐sinistrin clearance using this technique in unanesthetized rats should be made during the resting phase in order to minimally impact arterial pressure, heart rate, and activity.

Aside from anesthesia, stress and repeated blood sampling resulting in hypovolemia are also known to have a major impact on measurement of GFR. In this regard, many techniques adopted to assess renal function in unanesthetized rats involve immobilization of rats in restraint cages and/or blood sampling, often of an invasive nature, thereby disturbing the rats’ normal pattern of behavior and physiological and metabolic status. Such methods consequently impact arterial pressure and renal function (Chen and Herbert 1995; McDougall et al. 2000; Fitzner Toft et al. 2006). The results of the present investigation add important novel data to the existing body of evidence that measurement of FITC‐sinistrin clearance using a NIC‐Kidney device is minimally invasive and does not lead to gross changes in rat behavioral patterns. The size and attachment of the imager to the back of the rat did not appear to impede rat movement. Moreover, as documented above we showed that application of this technique during the resting period did not alter resting locomotor behavior, as observed during an equivalent time period at baseline. Furthermore, rats were allowed to move freely in an experimental chamber while data were collected, in the absence of any requirements for blood or urine collection. Of course, although we did not observe any gross changes in rat behavior it is possible that the GFR protocol may affect rat behavior in other ways not documented here. Furthermore, there still might be a degree of stress associated with the application of this technique. Measurement of corticosterone levels in rats undergoing this procedure might therefore be of interest in future studies.

It will also be of interest in future studies to assess the possibility of repeated measurements of transcutaneous FITC‐sinistrin clearance across shorter time periods (i.e., hours) than have previously been published. Generally speaking, a second measurement of FITC‐sinistrin clearance can be performed once the initial bolus of FITC‐sinistrin is cleared and the device signal is subsequently back to background fluorescence levels, at least using the described technique. In healthy rats, this typically occurs after 2–3 h of administration of the FITC‐sinistrin bolus that we administered. In comparison, in rats with significantly reduced kidney function this can take up to or greater than 24 h. Moreover, based on our own observations we would not recommend leaving the NIC‐Kidney device in place between extended GFR measurement periods since it is possible for rats to dislodge and damage the NIC‐Kidney device. Consequently, an additional anesthesia period would be required on the days in which the repeated measurements are made for reapplication of the device (including hair removal if required) and administration of FITC‐sinistrin.

In conclusion, our findings provide significant evidence that the described minimally invasive procedure for the transcutaneous assessment of FITC‐sinistrin clearance as a measure of GFR in unanesthetized, freely moving rats does not negatively impact on arterial pressure, heart rate or locomotor activity. Thus, based on our novel findings this method should be considered reliable for repeated measurements of GFR in rats.

## Conflict of Interest

None declared.
